# Case Report: Lymphocytosis Associated With Fatal Hepatitis in a Thymoma Patient Treated With Anti-PD1: New Insight Into the Immune-Related Storm

**DOI:** 10.3389/fonc.2020.583781

**Published:** 2020-12-14

**Authors:** Antonella Argentiero, Antonio Giovanni Solimando, Valentina Ungaro, Mariarita Laforgia, Sabino Strippoli, Dario Pinto, Antonio Negri, Simona Ferraiuolo, Alfredo Zito, Michele Guida

**Affiliations:** ^1^ Medical Oncology Unit, IRCCS Istituto Tumori “Giovanni Paolo II”, Bari, Italy; ^2^ Internal Medicine “G. Baccelli”, Department of Biomedical Sciences and Human Oncology, University of Bari, Bari, Italy; ^3^ Pharmacy Unit, IRCCS Istituto Tumori “Giovanni Paolo II”, Bari, Italy; ^4^ Rare Tumors and Melanoma Unit, IRCCS Istituto Tumori “Giovanni Paolo II”, Bari, Italy; ^5^ Radiology Unit, Ricerche Radiologiche “Maggialetti”, Molfetta, Italy; ^6^ Unit of Hematology and Cell Therapy, Laboratory of Hematological Diagnostics and Cell Characterization, IRCCS Istituto Tumori “Giovanni Paolo II”, Bari, Italy; ^7^ Pathology Unit, IRCCS Istituto Tumori “Giovanni Paolo II”, Bari, Italy

**Keywords:** thymoma, immune-microenvironment, immunotherapy, pembrolizumab, lymphocytosis, hepatitis, immune related adverse events

## Abstract

Recent advances in tumor immunotherapy have made it possible to efficiently unleash immune effectors, reacting against neoplastic cells. Although these approaches primarily aim to eradicate malignancy, immune-related adverse events (irAEs) often influence patients’ prognosis, constituting a new spectrum of side effects. Taking into account the typical microenvironment and the intricate equilibrium between the anti-tumor response and the immune cells, the thymoma constitutes a unicum in the immune-oncology field. We report a fatal immune-mediated adverse events’ storm in a thymoma patient treated with Pembrolizumab, leading to hepatotoxicity accompanied by lymphocytosis, thrombocytopenia, and thyroid dysfunction, unveiling a novel potential pathophysiological effect of immunotherapy. The clinical proficiency of the immune checkpoint inhibitors in thymoma patients warrants timely prevention and management of off-target consequences in order to optimize this promising therapeutic option. This case report describes a unique consequence of irAEs, emerging as a red flag warranting a multidisciplinary approach.

## Introduction

Thymic Epithelial Tumors (TETs) are thoracic malignancies with a low incidence in the worldwide population, that present with different clinical features and prognosis (1, 2). From the histopathological standpoint, thymomas have the greater incidence (highest value 0.68/100,000 in the 70–79-year-old age subgroup) compared to the thymic carcinoma (highest value 0.25/100,000 in the 70N74-year-old age subgroup). The 5-year overall survival (OS) rate are 90 and 55%, for thymoma and thymic carcinoma, respectively (1, 2).

**Figure 1:**
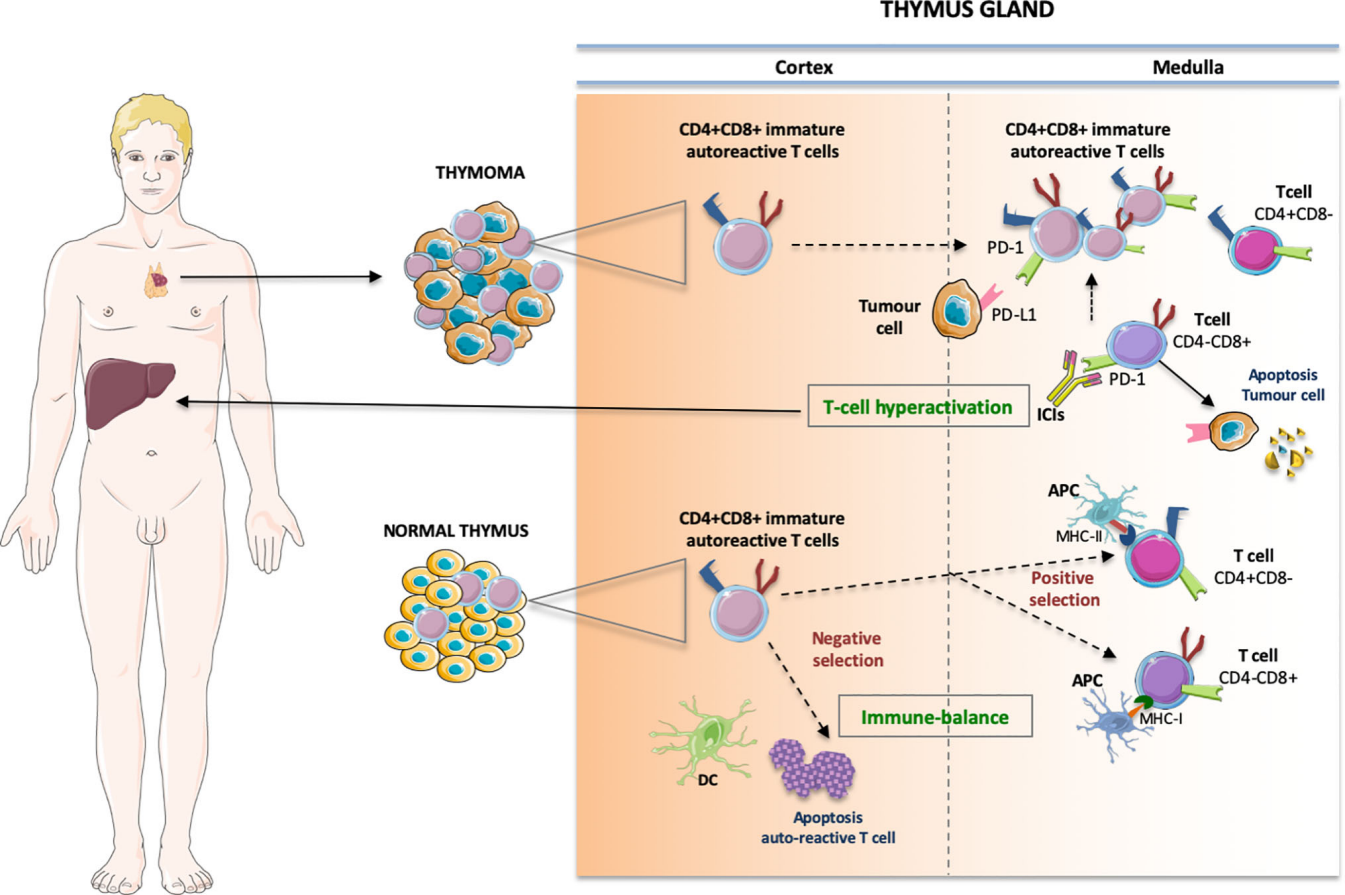
The pathophysiologic process that produces the immune-related storm in thymoma has not yet been fully clarified. Some possible vicious cycles involve enhanced T-cell activity against antigens present in the thymus and in the “innocent” bystander organs, whereby growing amounts of preexisting autoreactive T-cells are unleashed from the medulla, breaking the immune-equilibrium existing in the normal thymus. In particular, the immature CD4+CD8+ double positive cells move to single positive CD4+ helper T-cells (binding MHC class II) and CD8+ cytotoxic T-cells (binding MHC cIass I) involved in the adaptive immunity while a negative selection leads to apoptosis of self-reacting T clones. In thymic neoplasia the immature thymic lymphocytes may escape quality control by DC prompting T-cell hyperactivation and autoimmune manifestations. DC, dentritic cell; APC, antigen presenting cell; MHC, major histocompatibility complex.

Thymoma and thymic carcinoma share the origin in the thymic epithelium. The thymus is a complex immune organ, whose physiological role is to produce T-cells, involved in all processes of immune response comprising non-self reaction (3). Immature T-cells reach the thymus from the bone marrow through the blood stream. The precursors of *αβ* T-cells are deemed double-negative (DN). In the gland cortex, T-cell receptors (TCRs) CD4 and CD8 expression takes place. Therefore, CD4+CD8+ double positive (DP) T-cells carrying *αβ* TCRs recognize peptide-major histocompatibility (MHC) complexes, inducing the production of single positive (SP) CD4+ helper T-cells (MHCII) and CD8+ cytotoxic T-cells (MHCI), active in the adaptive immunity (4, 5). Conversely, a negative selection leads to apoptosis of self-reacting T clones (**Figure 1**).

Thymomas are generally associated with autoimmune disorders such as myasthenia gravis, pure red cell aplasia, and hypogammaglobulinemia, often requiring a full workup and multidisciplinary approach, comprising surgery, symptomatic therapy, immune suppression, and immunoglobulin administration (3, 6, 7). From a pathology standpoint, in thymomas T-cells are immature and express terminal deoxynucleotidyl transferase as phenotypic marker; conversely, thymic carcinomas are defined as tumors lacking immature T lymphocytes and never elicit an autoimmune disease. Therefore, the histological features and tumor microenvironment are key elements to distinguish thymomas from thymic carcinomas and, consequently, to tailor the most appropriate histotype-driven treatment (4, 8, 9).

Despite the demonstrated effectiveness of several agents against TETs, these often ultimately relapse, becoming resistant to drugs. Moreover, an optimal chemotherapeutic regimen in platinum-refractory TETs at advanced stages of disease has not yet been identified. Due to the rarity of the disease and the lack of significant randomized controlled trials, the available evidence has been obtained only from phase I to II trials of small and heterogeneous sample size, retrospective studies or published case reports (1).

Different studies showed that PD1/PDL1 inhibitors are effective in advanced TETs and contributed to the approval of Pembrolizumab for thymic carcinoma (10, 11). In the scientific literature, case series of Pembrolizumab or Avelumab also in thymoma subjects have been reported (10, 12). Despite an evident anti-tumor activity, immune-checkpoint inhibitors (ICIs) have displayed a critical immune-mediated toxicity profile; therefore, their safety represents an evolving landscape (10, 12, 13). Specifically, severe systemic ICI-related side effects in patients with thymoma have been described, including myocarditis, hepatitis, and myositis, highlighting the importance of careful patient selection while evaluating immunotherapeutic agents for treatment (**Supplementary Table 1**) (10, 12, 14, 15).

Given the promising anti-tumor activity and the peculiar thymic physiology within the immune system education, it is of paramount importance to identify potential factors able to predict ICI-related adverse events.

Herein, we report a case of fulminant immune-related hepatitis along with an autoimmune storm, heralded by a marked lymphocytosis, in a refractory patient affected by B2 thymoma treated with Pembrolizumab.

## Case Description

**Figure 2:**
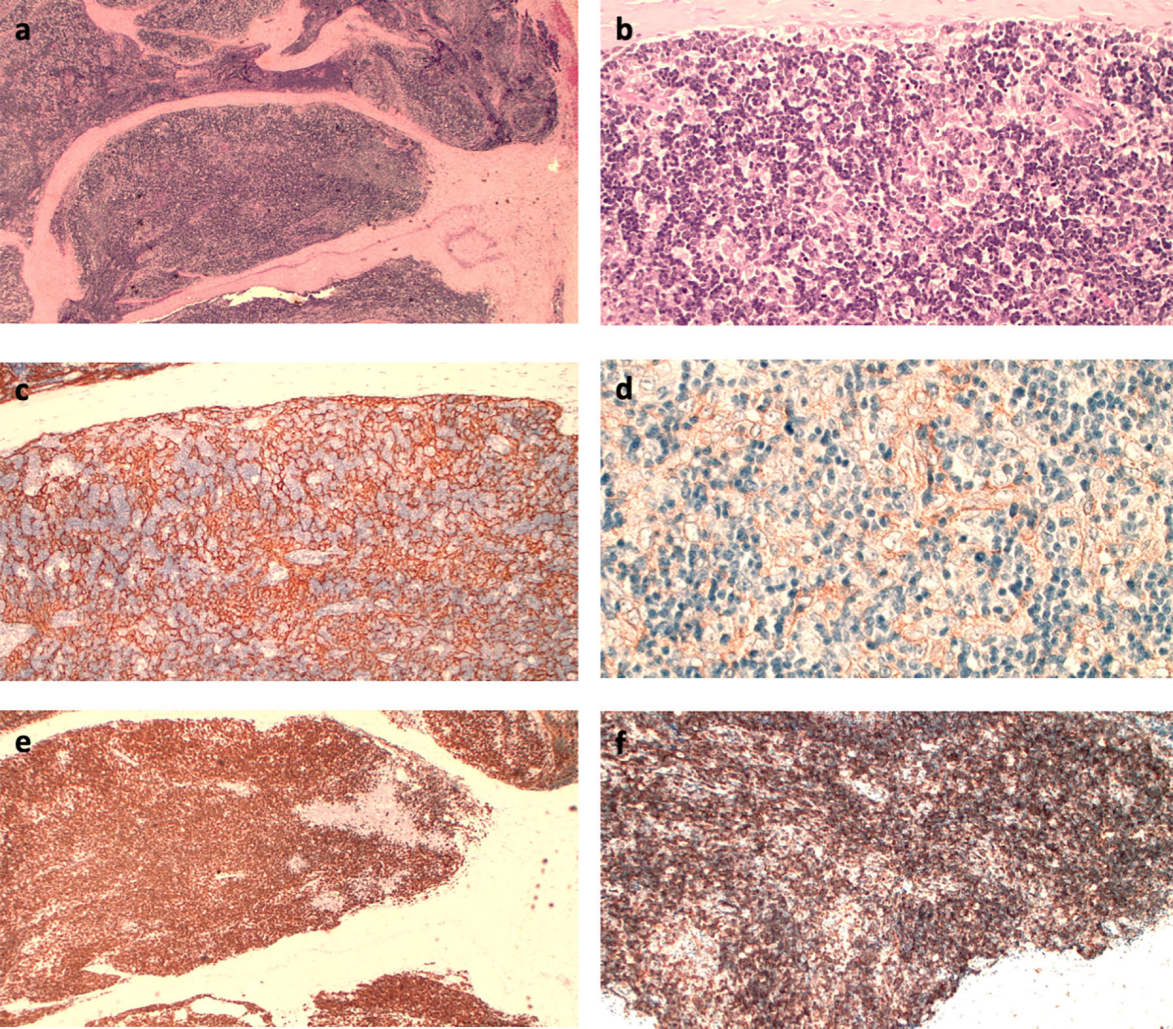
**(A)** Hematoxylin–eosin (HE) immunostaining (IHC): fibrous septa within the tumor separate the lymphoepithelial elements (4×); **(B)** HE IHC: aggregates of large epithelial cells with a clear cytoplasm interspersed between lymphoid cells (20×); **(C)** CK 19 IHC: dense epithelial cell network (10×); **(D)** PD-L1 IHC (Clone 22 C3): Membranous positivity of the epithelial cells (20×); **(E)** TdT IHC: lymphocytes consist predominantly of immature T-cells; **(F)** CD8 IHC: immune-positivity of CD8 on immature lymphocytes (10×).

A Caucasian 42-year-old male with metastatic thymomas received Pembrolizumab (2 mg/kg q21), after three lines of standard chemotherapy. Four years earlier, the patient had been found to have a massive anterior mediastinum mass and numerous pleural nodules during a workup for progressive dyspnea. Percutaneous pleural biopsy at that timepoint demonstrated CK19+, CK5/6+, EMA− and CK20− *versus* a CD99+, CD5+, TdT+, CD1a+ pattern in the epithelial and lymphoid components, respectively. Moreover, the tumor-infiltrating lymphocytes’ immunohistochemical phenotype demonstrated an immature T-cell population expressing TdT (presumed CD4, CD8 double positive) and PD-1 at baseline (**Figure 2**). Therefore, off-label medication with Pembrolizumab was required due to the failure of previous lines of chemotherapy and lack of other standard medications with proven effectiveness. At baseline, laboratory findings were unremarkable, except for a slight increase in absolute lymphocyte count (5,670/μl, normal range, 600 to 3,400/μl) and HBV seroconversion. The patient was admitted to hospital with fever and ecchymosis, 20 days after receiving his second dose of Pembrolizumab. Blood tests performed five days earlier revealed marked lymphocytosis, 11,600/μl; thrombocytopenia, 40,100/μl (normal range, 142,000 to 424,000/μl) and alterations of liver enzymes with increased aspartate amino transferase (AST) levels to 442 U/L (normal range up to 40 U/L) and alanine amino transferase (ALT) levels to 258 U/L (normal range 6−61 U/L). Initial workup upon hospitalization showed a further increase of lymphocytes, 24,310/μl (72% of 33,680/μl total white blood cells); thrombocytopenia, 31,000/μl, grade 4 hepatic toxicity with aspartate amino transferase (AST), 2,103 U/L increasing to 52 times normal range (up to 40 U/L), and ALT 714 U/L (normal range, 8 to 61 U/L); total and direct bilirubin, 18.19 mg/dl and 11.28 mg/dl, respectively (normal range up to 1.2 mg/dl and up to 0.3 mg/dl). Thyroid dysfunction was also shown, with FT4 >7.7 ng/dl (normal range 0.9 to 1.7 ng/dl) and TSH 0.013 mcUI/ml (normal range 0.3 to 4.2 mcUI/ml). Flow cytometric analysis was performed to study lymphocyte subpopulations. T-cells contained a predominant T-cytotoxic lymphocyte (CD3+, CD8+) population, along with low levels of natural killer T (NKT) cells and B-cells (**Supplementary Figure 1**).

**Figure 3:**
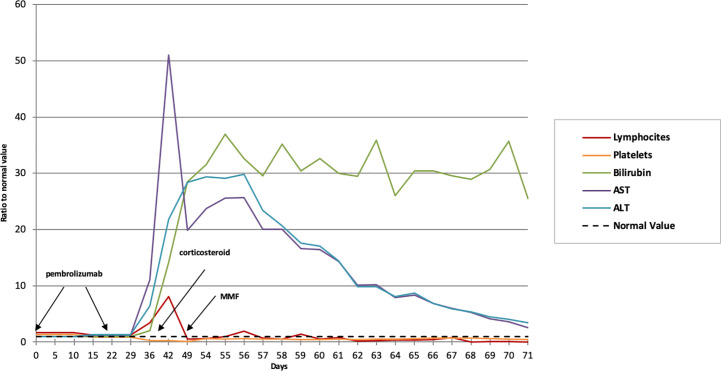
Biochemical and laboratory findings trend over time. The normal value (dotted line) refers to the unit, representative of the referral value to whom each fold change is referring to. MMF, mycophenolate mofetil.

**Figure 4:**
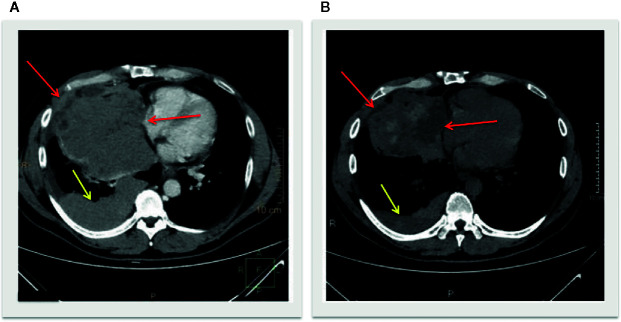
Longitudinal slices of computed tomography scans at baseline **(A)** and post treatment **(B)** showed a decrease of the mediastinum mass with intralesional hypondensity (red arrows) and pleural nodules (yellow arrows), defined as partial response according to iRECIST criteria.

Biochemical and laboratory findings paralleled the clinically evident liver failure course over time characterized by bruising, progressive, jaundice, changes in personality, aggressive behavior, sleeping disorders, and hepatic encephalopathy. Remarkably, early increased lymphocyte counts paralleled the exponential liver enzymes increase (**Figure 3**). Computed tomography scan revealed a partial response according to RECIST criteria, with a decrease of the mediastinum mass and pleural nodules (**Figure 4**).

On the assumption of an immune-related toxicity, a high-dose glucocorticoid regimen was administered (intravenous methylprednisolone 1 mg/kg/day, escalated up to 2 mg after 24 hours given the unsatisfactory laboratory improvement). Neither serologic evidence of acute active viral hepatitis nor ultrasound liver parenchyma alterations were detected, notwithstanding the fact that the patient harbored a seroconverted HBV positivity. Due to the severe thrombocytopenia, a liver biopsy could not be safely performed. Nonetheless, given the virologic history, entecavir 0.5 mg/die was administered. At 72 h after beginning steroid treatment, he started mycophenolate mofetil at 1,000 mg bis in die for progressive clinical deterioration. Selective bilirubin apheresis was required for worsening total bilirubin levels (44.36 mg/dl) and the appearance of psychomotor agitation. Despite these interventions, the patient developed a refractory hepatorenal syndrome and progressive fatal multisystem organ failure.

## Discussion

By re-educating the immune response against tumors, outstanding outcomes have been achieved in challenging oncologic scenarios; nonetheless, the clinical efficacy has often paralleled an uncontrolled immune activation, directed against off-target body functions and districts (16−17).

Herein, we report an early and fatal immune-mediated of high-grade adverse events in a thymoma patient treated with Pembrolizumab, characterized by dramatic lymphocytosis (CD8+ T-cell predominant), thrombocytopenia, hepatitis, and thyroiditis paralleling the manifest anti-tumor activity.

Recently, the US Food and Drug Administration (FDA) approved the use of Pembrolizumab in pre-treated thymic carcinoma, based on efficacy (overall response rate 22.5%) and safety data from a phase II study (11). Six patients out of 41 (15%) developed high grade irAEs, including four patients with hepatitis, three patients with polymyositis, and two patients with myocarditis. No deaths were reported (11). Pembrolizumab approval was restricted to thymic carcinoma subtype, due to pilot case series reporting immune-related toxicity in thymoma patients (**Supplementary Table 2**) (4).

Indeed, a phase II clinical trial by Cho et al. reported seven patients with thymoma (four pts B2, one pt B2/B3, two pts B3) treated with intravenous Pembrolizumab at a fixed dose of 200 mg every 3 weeks (10). The disease control rate (DCR) was 100%, with 28.6% of partial responses; the median PFS was 6.1 months (4.3 to 7.9 months), with a median number of 8 cycles (1–25). The major reason for treatment discontinuation was grade 3 or 4 irAEs occurring in five patients (71.4%), four of them within the first two administrations. Remarkably, two patients with toxicity and preexisting myasthenia obtained a partial response after only one cycle of treatment. The most frequently observed severe irAEs (G3-4) were autoimmune myocarditis (three patients); hepatitis (two patients); nephritis, colitis, thyroiditis, and conjunctivitis (one patient each). Six patients recovered from irAEs with high-dose corticosteroids, intravenous immunoglobulin, and other immunosuppressive agents. One death occurred due to cytomegalovirus superinfection during immunosuppressive treatment for severe hepatitis and colitis (10).

Similarly, Rajan et al. reported safety and efficacy of PD-L1 inhibition in a phase I clinical trial (12). Seven patients with pretreated thymoma (one pt B1, three pts B2 and three pts B3) received intravenous Avelumab at a dose of 10 to 20 mg/kg every two weeks. DCR was 86%. Partial response was observed in four patients (57%), most of whom developed irAEs after just one cycle of immunotherapy. Three of seven patients (43%) with thymoma suffered grade 3–4 irAEs, including shortness of breath, myositis, diarrhea, hyperkalemia, and asymptomatic elevated levels of liver transaminases. The irAEs were treated with corticosteroid therapy, achieving a rapid improvement of symptoms (12).

Literature data include sporadic additional case reports describing thymoma patients treated with ICIs and fatal multi-organ failure including myocarditis and myasthenia gravis, highlighting a critical scenario in terms of efficacy and toxicity (14, 15). Indeed, the anti-tumoral effect of ICIs often mirrors the onset of severe irAEs due to still-unexplored reasons.

Notably, in their case series Rajan et al. reported a correlation between the absolute lymphocyte count at baseline and the ICI response (12). Our report confirms the previous evidence, showing lymphocytes at baseline twofold higher than the normal limits. To our knowledge the case presented, with clinical and laboratory evidence offers the first description on an overt lymphocytosis within an immune-storm of adverse events and tumor shrinkage. This outcome warrants further statistically powered investigation in order to better define the real impact of increased lymphocyte absolute numbers in predicting irAEs and clinical response in thymoma patients.

These findings are substantially extended, providing a deeper insight into the pivotal role played by immune phenotyping in thymoma patients who are candidates for ICI.

As a paradigmatic condition for cancer development within a composite immune ecosystem, the thymic gland deserves a peculiar pathophysiological consideration as the soil that nurses malignant cells (3, 19). This emblematic crosstalk might justify the hazardous equilibrium between immune-tolerance and autoimmune brakes and suffice to explain the clinically explosive side effects with multi-system involvement (20).

Indeed, this storm of immune-mediated toxicity is a biological likelihood. The thymus is a lymphatic organ with a key role in the regulation of the immunological balance. Maintaining self-tolerance passes through the control of the differentiation and positive/negative selection of immature T-cells (21, 22). In physiological conditions, immature T lymphocytes migrate from the cortex to the medullary region, where the maturation process is completed following an accurate selection process. Thus, immature CD4−CD8− (double negative) is transformed into CD4+CD8+ (double positive) cells in the cortex region and can mature into either CD4+ or CD8+ single positive in the thymic medulla (23). The ravaged structure in thymic neoplasia hinders negative selection to eliminate autoreactive T-cells, and immature thymic lymphocytes may escape quality control by dendritic cells. Moreover, in neoplastic thymic epithelium, the production of regulatory T-cells is impaired, as well as the expression of auto-immune regulators (AIREs), a crucial issue in immune tolerance (24). Thus, in thymoma patients the function of self-tolerance is lost, prompting autoimmune manifestations. Moreover, the compromised expression of HLA-DR molecules in neoplastic thymocytes increases levels of HLA-A24 and HLA-B8, which are associated with an enhanced likelihood of autoimmune manifestations (25–27). Remarkably, the emerging correlation between a given HLA and irAEs highlights the potential relevance of the genetic background in predicting both immune side effects (28) and clinical efficacy (29).

In this precarious balance, PD-1/PD-L1 blockade upsets the immunological scenario. Blocking PD-1/PD-L1 reverts cancer immunotolerance, inducing disinhibition of effector T-cells and breakdown of peripheral tolerance against self-cells. Indeed, in physiological conditions, PD-1 restrains TCR-mediated positive selection through the PD-1/PD-L1 crosstalk (30, 31). Moreover, blocking PD1/PD-L1 signaling fuels the loss of inhibition of effector T-cells (Teff). Therefore, Teff can, in turn, better induce epithelial and endothelial thymic apoptosis and necrosis, whilst disabling immunological self-tolerance (32–35).

Thus, it is reasonable to envisage a vicious cycle feeding a bidirectional behavior of ICI-mediated effects that drive the on-tumor efficacy as well as the off-target side effects (**Figure 1**).

Consistent with previous findings, the detection of an immune tolerogenic microenvironment early on when starting thymoma patients on ICI-based treatment holds the potential to gain enhanced tumor shrinkage. However, an immune-mediated storm might also take place, mirroring the ICI off-target effects while hindering the PD-1 dependent immune-brake.

In this patient, the subclinical HBV seroconversion might have played an additional role in mediating an enhanced off-target irAE affecting the liver parenchyma. Notoriously, chronic HBV-viral infection induces a complex repertoire shaping the immune-infiltrate, which could have predisposed our patient to experience an increased immune flare mediated by the hepatic CD4+CD8+PD1+ milieu (36–38). Specifically, in HBV infected patients the increased regulatory T-cell (T_reg_) levels within the liver halt the effector function of CD4+ and CD8+ lymphocytes, sustaining an immune-suppressive milieu. Since PD1 is a crucial inhibitory receptor, its blockade might revert T-cell exhaustion, unleash the immune response and potentially lead to the hepatic damage (36–38). Despite the lack of clear guidelines driving the clinical approach to HBV seroconverted patients with thymoma, it is reasonable to retrospectively consider antiviral prophylaxis as a reasonable approach for ICI-treatment candidates (39–41). Indeed, the lesson learned from extensively investigated HBV reactivation in alternative clinical scenarios (42, 43) can be translated to the ICI-based approach, warranting a careful risk assessment at baseline and taking into consideration anti-viral prophylaxis.

Overall, clinicians should be vigilant against irAE in thymoma patients treated with ICIs, due to its prompt onset and fulminant progression. Other evidence has already shown thyroid immune-mediated damage to be an early side effect preceding and paralleling the autoimmune phenomena (44, 45). We confirm the above-mentioned clinical behavior. It is therefore tempting to speculate about the role of laboratory evaluation with full blood cell count, liver function assessment, and a full endocrine panel in making repeated indirect snapshots in order to avert the dynamic immune-related toxicity onset (46, 47). Thus, it is mandatory to better refine the druggable focuses, decreasing the incidence of undesired effects exerted by immune-system modulation and novel bullets striking the non-cancerous neighborhood. The rarity and heterogeneity of thymoma subtypes pose an objective difficulty in developing robust clinical trials to investigate efficacy and safety in this subset of TETs. In this framework, promising phase I–II clinical trials evaluating the safety and efficacy of ICIs in thymoma patients (NTC03076554, NCT03134118, NCT03295227) are ongoing.

## Ethics Statement

Written informed consent was obtained from the individual(s) for the publication of any potentially identifiable images or data included in this article.

## Author Contributions

AA, AS, and MG conceptualized the study. AA, ML, VU, AN, SF, SS, DP, and AZ contributed to the data curation. AS acquired the funding. MG supervised the study. AA, AS, ML, and VU wrote the original draft of the manuscript. MG wrote, reviewed, and edited the manuscript. All authors contributed to the article and approved the submitted version.

## Funding

This research project was also supported in part by the Apulian Regional Project “Medicina di Precisione” to AS.

## Conflict of Interest

The authors declare that the research was conducted in the absence of any commercial or financial relationships that could be construed as a potential conflict of interest.

## Supplementary Material

Supplementary Figure 1 Immunophenotypic characterization of lymphocytes in the peripheral blood. The lymphocyte population is identified as low SSC and bright CD45 (region “LYMPHOCYTES”) in the CD45 *vs* SSC dot plot. The percentages refer to the “LYMPHOCYTES” gated population. CD3+, T lymphocytes; CD19+, B lymphocytes; CD56+ CD16+ CD3−, NK lymphocytes; CD3+ CD56+, NKT lymphocytes; CD3+ CD4+, T helper lymphocytes; CD3+ CD8+, T cytotoxic lymphocytes. NK, natural killer.
