# Supplementary figures and images for: Case Report: Lymphocytosis Associated With Fatal Hepatitis in a Thymoma Patient Treated With Anti-PD1: New Insight Into the Immune-Related Storm
Source: Front Oncol. 2020 Dec 14;10:583781. doi: 10.3389/fonc.2020.583781 (PMC7768075; doi:10.3389/fonc.2020.583781)

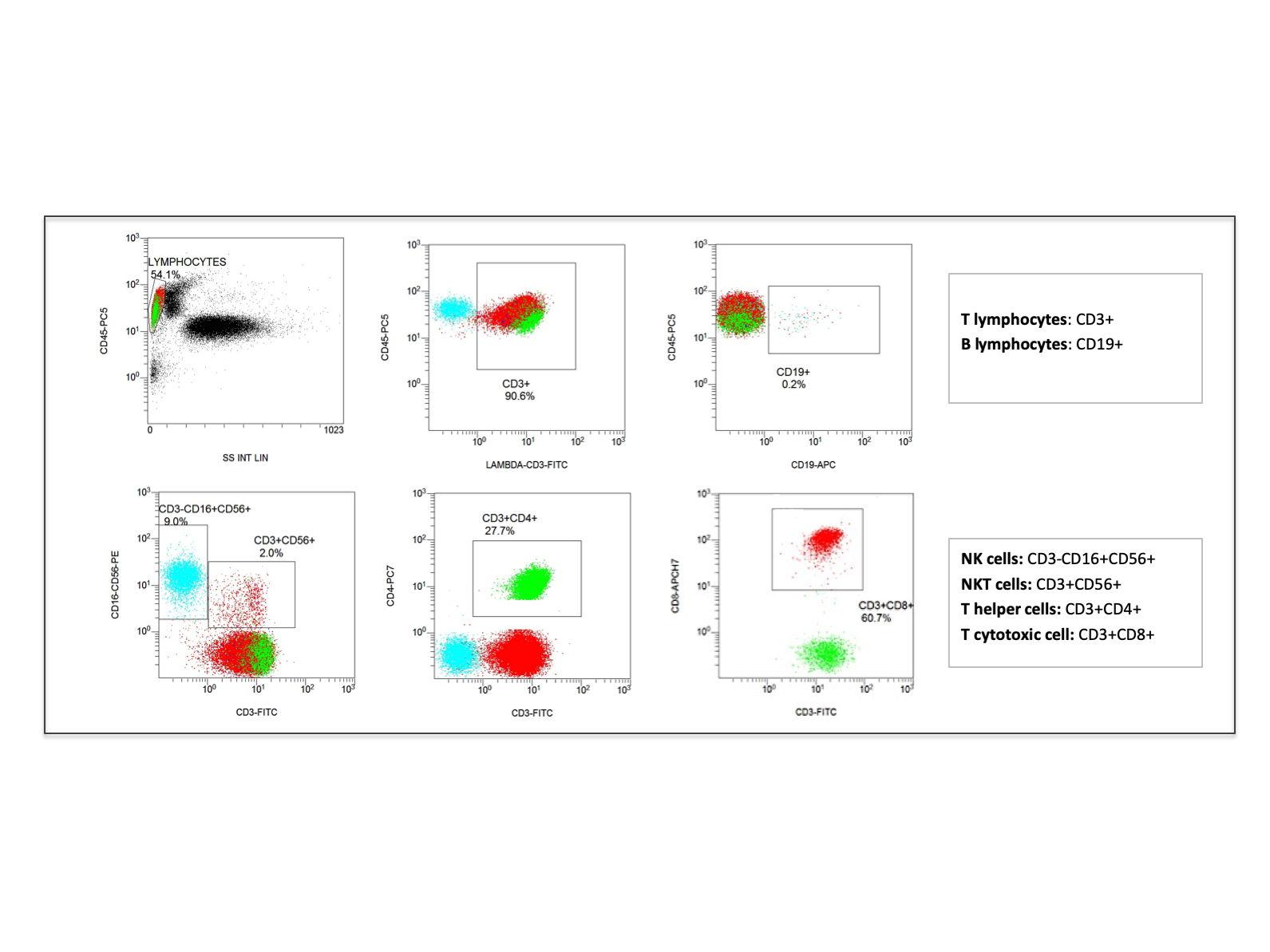

Supplement: Supplementary Figure 1 — Immunophenotypic characterization of lymphocytes in the peripheral blood. The lymphocyte population is identified as low SSC and bright CD45 (region “LYMPHOCYTES”) in the CD45 vs SSC dot plot. The percentages refer to the “LYMPHOCYTES” gated population. CD3+, T lymphocytes; CD19+, B lymphocytes; CD56+ CD16+ CD3−, NK lymphocytes; CD3+ CD56+, NKT lymphocytes; CD3+ CD4+, T helper lymphocytes; CD3+ CD8+, T cytotoxic lymphocytes. NK, natural killer. [file Image_1.tiff]
