# Supplementary material for: Case Report: Lymphocytosis Associated With Fatal Hepatitis in a Thymoma Patient Treated With Anti-PD1: New Insight Into the Immune-Related Storm
Source: Front Oncol. 2020 Dec 14;10:583781. doi: 10.3389/fonc.2020.583781 (PMC7768075; doi:10.3389/fonc.2020.583781)
Supplement: Supplementary file 2 [file Table_1.docx]

**Supplementary Table 1.** Efficacy and safety of PD1/PDL1 inhibitors for thymoma. irAEs, immune-related adverse events; n, number of patients; NA, not available.

| **References** | **Number of patients (*n*)** | **Immune checkpoint inhibitor** | **Response Rate**  **(%)** | **Grade 3/4 irAEs**  **(*n*)** | **Clinical Outcome of irAEs** |
| --- | --- | --- | --- | --- | --- |
| ***Cho et al.***  ***(12)*** | *n=7* | Pembrolizumab | 28.6% | - myocarditis (n=3)  - hepatitis (n=2)  - nephritis (n=1)  - thyroiditis (n=1)  - conjunctivitis (n=1) | Recovered (n=7);  one death due to super-imposed infection |
| ***Rajan et al.***  ***(13)*** | *n=7* | Avelumab | 28.5% | - shortness of breath (n=4)  - myositis (n=3)  - myocarditis (n=3)  - enteritis (n=1)  - hyperkaliemia (n=1)  - high transaminases (n=2) | Recovered (n=7) |
| ***Konstantina et al. (15)*** | *n=2* | Pembrolizumab | NA | - myocarditis (n=2)  - myositis (n=1)  - dermatitis (n=1) | Death  (n=2) |
| ***Hyun et al.***  ***(16)*** | *n=1* | Pembrolizumab | NA | -myocarditis (n=1)  - myasthenia gravis crisis (n=1)  - hepatic dysfunction (n=1) | Death  (n=1) |
