# Supplementary material for: Case Report: Lymphocytosis Associated With Fatal Hepatitis in a Thymoma Patient Treated With Anti-PD1: New Insight Into the Immune-Related Storm
Source: Front Oncol. 2020 Dec 14;10:583781. doi: 10.3389/fonc.2020.583781 (PMC7768075; doi:10.3389/fonc.2020.583781)
Supplement: Supplementary file 3 [file Table_2.docx]

**Supplementary Table 2.** Laboratory Tests from the first administration of Pembrolizumab to the fatal event. LY, lymphocyte; PLT, platelets; aspartate amino transferase (AST), aspartate amino transferase (AST).

| **Frequency of lavoratory tests** | **LY**  **(x10^3^/μL)** | **PLT**  **(x10^3^/μL)** | **Direct bilirubin**  **(mg/dL)** | **AST**  **(U/L)** | **ALT**  **(U/L)** |
| --- | --- | --- | --- | --- | --- |
| First administration: Day 1 | 5,67 | 188 | 0,8 | 38 | 34 |
| Second administration: Day 22 | 4,28 | 119 | 0,9 | 50 | 54 |
| Hospitalization: Day 42 | 11,6 | 40 | 11,28 | 442 | 258 |
| Start Cortison Therapy Day 49 | 27,37 | 38 | 16,99 | 2041 | 871 |
| Day 54 | 1,76 | 17 | 34,15 | 796 | 1137 |
| Day 56 | 3,27 | 75 | 44,36 | 1022 | 1164 |
| Day 57 | 6,64 | 81 | 39,16 | 1027 | 1191 |
| Day 58 | 2,34 | 71 | 35,4 | 801 | 937 |
| Day 59 | 1,89 | 70 | 42,22 | 800 | 826 |
| Day 60 | 4,71 | 62 | 36,49 | 664 | 703 |
| Day 62 | 2,73 | 70 | 36,03 | 573 | 578 |
| Day 65 | 1,23 | 73 | 31,19 | 317 | 324 |
| Day 70 | 0,24 | 85 | 36,79 | 165 | 178 |
| Death: Day 72 | 0,12 | 61 | 30,59 | 103 | 135 |
